# Development of *In-Situ* Spray for Local Delivery of Antibacterial Drug for Hidradenitis Suppurativa: Investigation of Alternative Formulation

**DOI:** 10.3390/polym13162770

**Published:** 2021-08-18

**Authors:** Yoke Lan Wong, Manisha Pandey, Hira Choudhury, Wei Meng Lim, Subrat Kumar Bhattamisra, Bapi Gorain

**Affiliations:** 1 School of Pharmacy, International Medical University, Bukit Jalil, Kuala Lumpur 57000, Malaysia; WONG.YOKELAN@student.imu.edu.my; 2 Department of Pharmaceutical Technology, School of Pharmacy, International Medical University, Bukit Jalil, Kuala Lumpur 57000, Malaysia; weimeng_lim@imu.edu.my; 3 Centre for Bioactive Molecules and Drug Delivery, Institute for Research, Development and Innovation, International Medical University, Kuala Lumpur 57000, Malaysia; 4 Department of Life Sciences, School of Pharmacy, International Medical University, Bukit Jalil, Kuala Lumpur 57000, Malaysia; bhattamisra@yahoo.co.in; 5 Center for Drug Delivery and Molecular Pharmacology, Faculty of Health and Medical Sciences, Taylor’s University, Subang Jaya 47500, Malaysia; bapi.gn@gmail.com; 6 School of Pharmacy, Faculty of Health and Medical Sciences, Taylor’s University, Subang Jaya 47500, Malaysia

**Keywords:** hidradenitis suppurativa, in situ spray, anti-microbial, sustained release, thermoresponsive gel, topical application

## Abstract

Hidradenitis suppurativa (HS) has been considered an orphan disease with limited treatments available. The available topical treatment for this condition is clindamycin lotion; however, short retention and frequent application are the main setbacks. Thus, the present study aimed to attain an optimized antibacterial in situ spray formulation for the hidradenitis suppurativa skin condition, which gels once in contact with the skin surface at around 37 °C and possesses bioadhesion as well as sustained-release properties of the incorporated drug. Different concentrations of thermo-reversible gelling polymer, Pluronic F-127, were investigated along with the selected bioadhesive polymers, HPMC and SA. The optimized formulation F3 consisting of 18% Pluronic F-127 with 0.2% HPMC and 0.2% SA was characterized based on various physicochemical properties. The gelation temperature of F3 was found to be 29.0 ± 0.50 °C with a gelation time of 1.35 ± 0.40 min and a pH of 5.8. F3 had the viscosity of 178.50 ± 5.50 cP at 25 °C and 7800 ± 200 cP at 37 °C as the gel set. The optimized formulation was found to be bioadhesive and cytocompatible. Cumulative drug release was 65.05% within the time-frame of 8 h; the release pattern of the drug followed zero-order kinetics with the Higuchi release mechanism. The average zone of inhibition was found to be 43.44 ± 1.34 mm. The properties of F3 formulation reflect to improve residence time at the site of application and can enhance sustained drug release. Therefore, it could be concluded that optimized formulation has better retention and enhanced antimicrobial activity for superior efficacy against HS.

## 1. Introduction

Skin diseases are vastly affecting teenagers and adults around the world. It has been viewed as a major problem as there these skin diseases can affect patients’ everyday life, whereby their physical and social lives are impacted, with a great impact on the psychological aspects of the people around them [1]. Hidradenitis suppurativa (HS) is a chronic inflammatory disease of the skin that affects around 1% of the general population [2] and it has an estimated prevalence of 1 to 4% [3]. It has been noticed that lack of awareness led to this disease being misdiagnosed often, especially in the early stages, or diagnosed late, with an average of 7 years [3,4]. HS can present in many people, where an appropriate early recognition and diagnosis are important to help patients understand the situation and ensure prompt disease management [5,6] to avoid an increase in the development of patients’ secondary psychiatric disorders, for instance, anxiety and depression, that could eventually lead to a higher risk of suicide [2]. The progression of the disease is rapid during the period of misdiagnosis or late recognition, where the disease leads to minor inflammations until the destruction of the skin tissues. The only treatment for such conditions is to undergo surgical removal of the skin on the infected site [4]. Surprisingly, a recent report revealed that the unemployment rate of the patients diagnosed with HS is 25.1% as compared to the control population at 5.9% [4].

HS has been considered as an orphan disease with limited treatments available [3]. This is not an infectious disease, however, the presence of bacteria plays important role in the pathophysiology of the disease. *Staphylococcus* is predominantly found in the abscess and nodule of HS, where the untreated disease condition may compromise the quality of life [7]. The disease mainly affects the intertriginous skin, such as at axillary, perianal, as well as inguinal sites [4]. The severity of the condition is commonly classified by using the Hurley staging system. There are three stages for HS: the first stage is the formation of single painful inflamed nodules or abscesses; it is the most important stage that if adequate treatment or therapy was given, there is a high chance that the condition would recur or build fistulae in which secretion of malodorous purulent would occur. That is how to identify stage II of HS. Stage III is considered when the inflammation leads to skin destruction; multiple abscesses and interconnected fistulae could be observed along with stiff hypertrophic scars [4]. In addition to the lifestyle advice and psychosocial modifications for HS, there is a range of topical and systemic therapies that can be used, depending on the conditions of the disease. Clindamycin is known as the sole medication in a topical form that shows improvement in superficial lesions of HS with Hurley stage I or II after 2 to 3 months of usage [5]. There are topical solutions and gel forms of clindamycin preparations are available commercially; however, retention time and contamination during multiple applications of the commercial formulations are the major setbacks. There has yet to be a consideration on the design of the formulation where the treatment of early-stage HS could be more effective by enhancing bioadhesive property and retention of the formulation at the site of action. In this regard, using an in situ gel with a prolonged contact time and a sustained drug release effect could improve the therapeutic efficacy as the inflammation is mainly found at the epidermis and upper dermis skin layer [8]. In situ gels are viscous liquids which have a unique characteristic of undergoing sol-to-gel transitions by the influence of various stimuli, namely pH, temperature, and electrolytes [9]. In this study, the focus of sol-gel transition was made on the temperature-dependent factors, where the conversion of sol-to-gel conversion was projected to the stimuli of skin temperature. As the in situ gel would be delivered onto the skin by spray form, this will hold a few advantages over other dosage forms: (i) reduction in the frequency of administration, (ii) effective technique to prevent the potential risk of cross-contamination, and (iii) improvement in patient compliance and comfort [10]. Therefore, the aim of this present experiment was to develop and optimize an in situ spray possessing good bioadhesion at the skin condition with the sustained-release property of clindamycin. Therefore, the optimized formulation would exert a superior antimicrobial efficacy against the pathogenic bacteria of HS with good compatibility of the formulation to the skin. In a broader consideration, the in situ spray was formulated to establish a biocompatible, environmental and skin friendly formulation.

## 2. Materials and Methods

### 2.1. Materials

Pluronic F-127(12600 g/mol) and sodium alginate (SA, M/G ratio 1.56) were purchased from Sigma-Aldrich. Germany. Hydroxypropyl methylcellulose (HPMC K4M), carboxymethyl cellulose (CMC) (average Mw ~90,000), xanthan gum, sodium dihydrogen phosphate, disodium hydrogen phosphate, 70% ethanol (EtOH), methylene blue dye, TSA, DMSO, MTT reagent, Dulbecco’s Modified Eagle Medium (DMEM), ultrapure water (Sartorius H2OPRO-VF-T), clindamycin hydrochloride (pharmaceutical secondary standard), fetal bovine serum (FBS), and Penicillin-Streptomycin were purchased from Life Technologies (New York, NY, USA). Human keratinocyte cell lines (HaCaT) were obtained from CLS-Cell Line Services (Eppelheim, Germany). *Staphylococcus aureus* (ATCC 6538) for microbiological assay was procured from the Microbiology Research Laboratory, International Medical University, Kuala Lumper, Malaysia. The fresh pigskin, marketed 1% clindamycin topical solution, and marketed 1% clindamycin topical gel were purchased from the local market.

### 2.2. Methodology

#### 2.2.1. Formulation of In Situ Gelling System

The formulation was prepared by the cold method [11] with the simple dissolution of polymers. Firstly, the weighed amount of different concentrations of Pluronic F-127 ranging from 14 to 22% were pre-dissolved with 100 mL 4 ± 2 °C cold ultrapure water separately and kept overnight in the refrigerator at a constant temperature of 4 °C for at least 10 to 12 h. After it was observed that they had been well-dissolved with clear solutions, 10 mL of each formulation was transferred to empty 250 mL beakers to test for gelation temperature and gelation time.

Gelation temperature was determined by immersing half the beaker containing 10 mL formulations into a water bath with the temperature kept constant at 37.0 ± 0.20 °C until no liquid flowed when the beaker was tilted to 90°. The time taken for the gel to form was then recorded at that gelling point. A preliminary study was carried out to determine the bioadhesive polymers suitable to be used for further development of formulation. HPMC, methylcellulose (MC), carboxymethyl cellulose (CMC), xanthan gum, and SA were considered. Each bioadhesive polymer in different concentrations (0.05%, 0.1%, 0.2%, and 0.4%) was mixed with 10 mL of the selected concentration of Pluronic F-127. The formulations were observed visually for the texture of both the solution at room temperature at approximately 25 °C, as well as the gel state after immersing in the water bath of 37.0 ± 0.20 °C.

#### 2.2.2. Characterization of In Situ Gelling Formulation

The developed formulation was then taken to undergo different tests for the characterization based on physicochemical properties such as gelation temperature, gelation time, pH, viscosity, spray pattern, bioadhesion, and ex vivo retention [11,12]. The optimized formulation determined by these properties would then be taken for further tests, such as the in vitro drug release test, antimicrobial test, cytotoxicity test, as well as to observe the cell morphology [11,12].

#### 2.2.3. Measurement of pH

The pH of all formulations was determined using a digital pH meter. Buffers of pH 4.0 and 7.0 were used for the calibration before the measurements [13].

#### 2.2.4. Measurement of Viscosity

Viscosities were measured using a Brookfield viscometer (DV2T, Brookfield Engineering Laboratory, Middleborough, MA, USA) at two different temperatures of 25 °C and 37 °C [11,14], at the speed of 100 rpm using spindle numbers LV 02 and LV 04, respectively.

#### 2.2.5. Spray Pattern

A small drop of methylene blue dye was added to the developed formulation and mixed well. The formulation was then loaded into a spray bottle and sprayed onto an A4 size white paper for visual observation.

#### 2.2.6. Ex Vivo Bioadhesion Test

An ex vivo bioadhesion test was performed by determining the force required to detach the fresh pigskin from the formulations applied using a modified physical balance [11,12,13]. A piece of 4 cm square-sized fresh pigskin was fixed under one pan of the balance; a total of 0.5 g of the developed formulation was placed on the inverted beaker acting as a stand. The fresh pigskin was lowered to adhere to the formulation. A total force of 0.5 N was applied for 20 s to ensure the standard condition allowing bioadhesion to occur. The force was removed and water was added slowly on the other side of the pan until the detachment was observed. The total amount of water required was weighed and recorded. After every measurement, the fresh pigskin was gently rinsed with phosphate buffer pH 7.4 and left to settle for 5 min before the next formulation testing. Fresh skin was replaced before carrying out the second as well as the third replications [15].

#### 2.2.7. Ex Vivo Retention Test

A half-A4 paper size of fresh pig skin wrapped around a 500 mL beaker was placed on a hot plate with the temperature kept constant at 37.0 ± 0.2 °C. The temperature of the fresh pigskin was measured and recorded. The selected formulations were sprayed onto the skin, visually examined, and compared the retention effect immediately and after 2 h.

#### 2.2.8. Preparation of Clindamycin In Situ Spray

After the formulation has been optimized, 1% of clindamycin was added and stirred gently with a magnetic stirring bar for 5 min to dissolve. The clindamycin was found to be water soluble and dissolved easily with the selected formulation. The optimized formulation was ready for in vitro drug release test and antimicrobial test.

#### 2.2.9. Preparation of Standard Curve for Clindamycin

A total of 10 mg of clindamycin was dissolved in 50 mL of ultrapure water to make a stock solution with a concentration of 200 μg/mL. The standard solutions for the standard curve were prepared by taking 1, 2, 3, 4, and 5 mL from the stock solution, respectively. Each volume was then made up to 10 mL solution using the volumetric flask. The solutions were filtered with filter paper. Absorbance values were recorded at 210 nm [16] using a UV-Vis spectrophotometer (Shimadzu UV-1800).

#### 2.2.10. In Vitro Drug Release Test and Release Kinetics

A buffer solution of pH 7.0 was prepared using 7.744 g of di-sodium hydrogen phosphate and 2.913 g sodium di-hydrogen phosphate with 500 mL of ultrapure water. A total of 400 mL of water was measured and placed on the magnetic stirrer. The weighed 7.744 g of disodium hydrogen phosphate was added into the beaker. The weighed 2.913 g of sodium di-hydrogen phosphate was then added into the beaker. A necessary amount of HCl was used to adjust the pH of the buffer solution to obtain pH 7.0, and the volume was adjusted to 500 mL.

A dialysis membrane of 25 mm (W) × 16 MM (Dia.) × 2.01 mL/cm with 8000 to 14,000 KD was pre-soaked in a buffer solution of pH 7.0 for 24 h. A measured volume of the optimized formulation (2 mL) was loaded into the dialysis bag before placing it into a 100 mL beaker containing 50 mL of phosphate buffer solution (pH 7.0), which was used as the release medium. Simultaneously, 2 mL of a control formulation without drugs was also tested, along with 1% of clindamycin in ultrapure water, marketed 1% clindamycin topical solution, and marketed 1% clindamycin in topical gel. The beakers were kept in the water bath shaker abd maintained at 60 rpm, with the temperature kept constant at 37 °C. Samples of 2 mL from the middle position of each beaker were withdrawn at definite time intervals for the absorbance measurement using UV-Visible spectrophotometer at 210 nm. An equal amount of fresh medium (pH 7.0 phosphate buffer solution) was replaced immediately after the withdrawal to maintain the initial volume. The experiment was continued for 8 h [11,12,13]. Various mathematical models were used on the data obtained from the in vitro drug release test to assess the drug release kinetics [17].

#### 2.2.11. Antimicrobial Test

The antimicrobial test was performed using the disc diffusion method [18]. Stock culture of *Staphylococcus aureus* stored at the freezer of −70 to −80 °C was retrieved and used as the test microorganism. The test samples prepared for the test were blank, plain optimized formulation, marketed topical solution (1% clindamycin), 1% standard clindamycin solution, and optimized formulation with 1% clindamycin. A total of 45 g of TSA (Trypticase Soy Agar) was dissolved in 1 L of ultrapure water to make the TSA solution by boiling in the water bath. All the materials required, including TSA solution, were autoclaved at 121 °C for 15 min before the experiment. *Staphylococcus aureus* was cultured for 24 h in a TSA agar medium at 37.0 ± 0.2 °C. The optical density (OD) of the growth was determined at 625 nm. Each antibiotic disc contained 15 μL of test samples. The cultured cells were then spread evenly onto the TSA agar dish using sterile cotton buds; the discs (diameter 6 mm) were placed at the centre of each dish and kept incubated at 37.0 ± 0.2 °C for another 24 h. The growth of bacteria *Staphylococcus aureus* was observed, and the zone of inhibition was measured for each sample in triplicate.

#### 2.2.12. Cytotoxicity Test

The HaCaT was cultured in Dulbecco’s Modified Eagle Medium (DMEM) along with 10% FBS and 1% penicillin-streptomycin [19]. A total of 5000 cells per well were seeded in a 96-well plate. The solution was incubated for 24 h before treating, with the optimized formulation at concentrations of 1.25 to 10 mg/mL. The test samples were incubated for 72 h. MTT reagent was added and incubation continued for another 4 h. The medium was removed and DMSO was added to dissolve the formazan crystals formed. The plate was read at 570 nm and 630 nm using a microplate reader. The treated cells at concentrations of 1.25, 2.5, 5, and 10 mg/mL were observed under the microscope [20] (Nikon Eclipse Ti-U Inverted Microscope) at 4× after 72 h of incubation. A comparison was made visually between the control and the treated cells.

#### 2.2.13. Statistical Analysis

The data represent the mean of three replicates ± standard deviation (SD) and were analyzed using ANOVA with the post-hoc Tukey test. Differences between means were considered significant at a *p*-value < 0.05 [21].

## 3. Results

### 3.1. Formulation of In Situ Gelling System

The gelation temperature and gelation time of Pluronic F-127 solution in different concentrations ranging from 14% to 22% were tested, as mentioned in Table 1. In the water bath with a temperature maintained at 37.0 ± 0.2 °C, the timer started once the 250 mL beaker containing 10 mL of the formulation was half immersed in it, with the starting temperature of 15 °C. The dispersions with 14% Pluronic F-127 and 16% Pluronic F-127 did not show any gelation. The solutions remained in liquid form even after 4 min. The gelation was observed in the formulations with Pluronic F-127 of 17% onwards, with a trend of decreasing in both temperature and time for the complete gel formation. The beaker was tilted 90° to ensure no flowing of the solution when the final temperature and time were recorded. At 17% of Pluronic F-127, the average gelation temperature and time were recorded as 34.0 ± 1.08 °C and 3.21 ± 0.01 min, respectively; simultaneously, 18% Pluronic F-127 dispersion resulted at 31.2 ± 0.58 °C and 2.22 ± 0.02 min, respectively; a total of 19% Pluronic F-127 dispersion resulted at 29.0 ± 1.00 °C and 1.43 ± 0.02, respectively; a total of 20% dispersion resulted at 28.0 ± 0.50 °C and 1.25 ± 0.05 min, respectively; a total of 22% dispersion resulted at 26.7 ± 0.76 °C and 1.12 ± 0.10 min, respectively.

Based on the results, Pluronic F-127 at the concentration range of 17–22% was selected to observe the impact of the bioadhesive polymer at a constant concentration. HPMC and SA were selected to test for the effect of combining bioadhesive polymers in a sol-gel formulation. Three different concentrations of HPMC and SA at 0.1%, 0.2%, and 0.4% were dispersed separately to the selected Pluronic F-127 formulation, with the gelation temperature and gelation time that met the set target prior to the experiment kept as the highest. Firstly, the concentrations of HPMC and SA were kept constant at 0.2% each, to be tested with Pluronic F-127 at different concentrations from 17% to 22%. At 17% Pluronic F-127, the average gelation temperature and time with 0.2% HPMC were recorded at 32.3 ± 0.30 °C and 2.35 ± 0.30 min, while with 0.2% SA, no gelation was observed (Table 2). Comparing results depicted in the data of Table 1 and Table 2, it was evident that the addition of the bioadhesive polymer reduced the gelling temperature and time significantly. Formulation with 17% Pluronic showed a significant difference when compared with formulation with 18%. Although the 19% Pluronic F-127 may take slightly less time for the gel formation, there is no significant difference with the 18% Pluronic F-127 formulation with the bioadhesive polymer, as analyzed by ANOVA with the post-hoc Tukey test. Therefore, the formulation with 18% Pluronic F-127 has been chosen as the base formulation for further development and optimization.

While keeping the concentration of Pluronic F-127 solution constant at 18%, further tests were then carried out to observe and determine the optimum concentrations of HPMC and SA, suitable for the combination of both the bioadhesive polymers selected with the 18% Pluronic F-127 solution by the results of gelation temperature and gelation time, as well as a visual inspection to obtain an optimized sol–gel formulation that fits the characterization of an in situ spray. In this regard, HPMC at 0.1% and 0.2% were then chosen to combine with 0.1% and 0.2% of SA, respectively, in separate 10 mL Pluronic F-127 solutions. Four sets of samples with different concentrations of the selected bioadhesive polymer were well-dissolved in the base formulation: 0.1% HPMC with 0.1% SA, 0.1% HPMC with 0.2% SA, 0.2% HPMC with 0.1% SA, and 0.2% HPMC with 0.2% SA were obtained. The average gelation temperature and gelation time for the combination of 18% Pluronic F-127 with HPMC and SA of concentrations 0.1%, 0.2%, and 0.4% were displayed in Table 3. Viscosity and spray ability are the major characteristics in the selection process of the formulation. As the HPMC and SA at a concentration of 0.4% each showed relatively high viscosity, the total concentration of HPMC and SA beyond 0.4% within the formulation would not be considered; only 0.1% and 0.2% of HPMC and SA were used for the next step of optimization. It was observed that on the increment of bioadhesive polymer concentration, gelation time, and temperature were also decreased.

The average gelation temperature and gelation time for the optimization of Pluronic F-127 in combination with the two bioadhesive polymers were recorded. As indicated in the results, the combination of both the polymers at different concentrations (0.1 and 0.2% *w*/*v*) did not show a significant effect on gelation time and temperature (Table 3). The gelation temperature and gelation time of the optimized sol–gel formulation chosen were at an average of 29.0 ± 0.50 °C and 1.35 ± 0.40 min. Thereby, the optimized formula of the in situ gel formulation consisted of the combination of 18% Pluronic F-127 with 0.2% HPMC and 0.2% SA.

### 3.2. Viscosity

**Figure 1:**
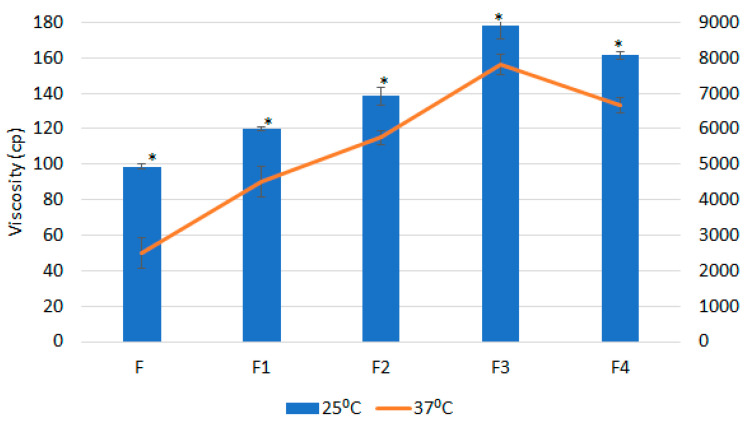
Viscosity (cP) of formulations at 25 °C and 37 °C; data presented as mean ± SD; *n* = 3; * indicates significant change in viscosity at 37 °C when compared to the corresponding viscosity at 25 °C (*p* < 0.05).

The viscosities of the samples (F, F1, F2, F3, and F4—composition given in Table 4) were recorded in triplicate at two different temperature conditions of 25 °C and 37 °C. At 25 °C, the average viscosity for the F3 was at 178.50 ± 5.50 cP, while at 37 °C the average viscosity was calculated 4300% higher (7,800 ± 200 cP). Similar patterns were observed with F1, F2, and F (Figure 1). These results also indicate that the addition of the bioadhesive polymer significantly enhances the viscosity at both temperatures. However, this difference was higher when the combination of bioadhesive polymer was compared to single bioadhesive polymer; additionally, this drastically increased in viscosity while changing the temperature-depicted sol–gel transition of formulation at body temperature.

### 3.3. Ex Vivo Bioadhesion Test

Five samples (F, F1, F2, F3, and F4) were tested in triplicates, and the calculated results of the detachment force were depicted in Table 4. These results supported that formulation with combinations of bioadhesive polymers significantly enhanced detachment force when compared to F, F1, and F2 formulations. Moreover, the percentage of the bioadhesive polymers impacted on bioadhesion property.

### 3.4. Ex Vivo Retention Test

On visual inspection, the F3 formulation showed a relatively faster gelling effect after spraying onto the fresh pigskin as compared to F1 and F2, in which the temperature was retained at 37.0 ± 0.20 °C, representing the body skin temperature, mainly focused on mimicking the axilla. The retention effect was also observed after 2 h of spraying the formulation, which confirmed the retention of the formulation at the site of application without any change in physical state.

Based on gelation temperature, viscosity, bioadhesion test, and ex vivo retention test, formulation F3 was considered for further investigations.

### 3.5. pH of the In Situ Gelling Formulation

The pH value of the optimized sol–gel formulation was recorded at pH 6.3. After the loading of 1% clindamycin into the formulation, it was recorded at pH 5.8.

### 3.6. Spray Pattern

**Figure 2:**
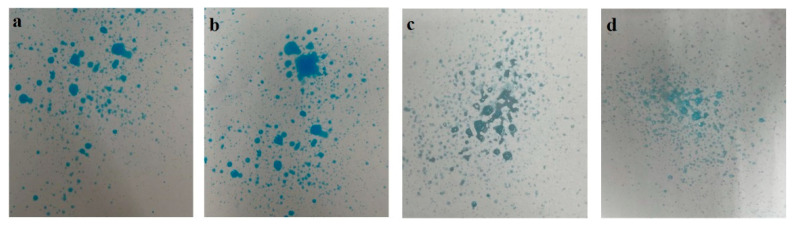
Spray patterns of the formulation (**a**) F, (**b**) F1, (**c**) F2, and (**d**) F3.

Visual observation was conducted for the spray pattern of the formulations (F, F1, F2, F3). There has been consideration whether spray form would contribute to the air pollution; the spray pattern of the developed formulations showed that most of the dispensed formulations appeared as larger and heavier forms of droplets (Figure 2). There should be no worry regarding the pollution as the sprayed droplets would not be in the form of aerosol.

### 3.7. In Vitro Drug Release Test

**Figure 3:**
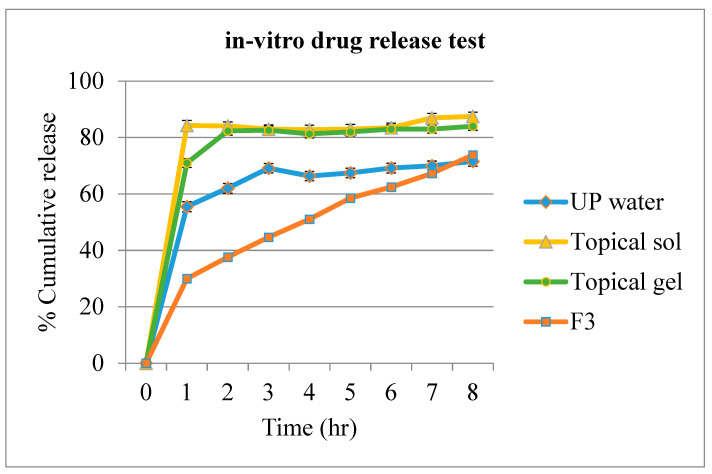
Presentation of cumulative release (%) of clindamycin from different formulations (Data presented as mean ± SD; *n* = 3).

Using the standard curve, the concentrations of the released drug at different time points were calculated. Simultaneously, the percentage cumulative drug release of the different samples was calculated. Over the 8 h of drug release, results showed 73.8% cumulative release of the drug from the optimized in situ gel formulation of clindamycin. From Figure 3 observed, the curve showed a stable continuous release pattern.

The drug release tests have also been performed for the 1% clindamycin in ultrapure water, marketed 1% clindamycin topical solution, and marketed 1% clindamycin topical gel. The cumulative release of the drug from the standard 1% clindamycin in ultrapure water was found to be 69.14% within 3 h. The marketed topical solution resulted in a burst release of 84.30% within 1 h, whereas a total of 87.5% release within 3 h was observed for the topical gel product. Both the marketed products showed an almost similar pattern of degradation after the release.

### 3.8. Drug Release Kinetics

Various mathematical models for drug release kinetics were used in an attempt to fit the results obtained from the in vitro drug release test. The results based on the regression correlation (R-squared) of the optimized formulation (F3) showed 0.8939 for zero-order, 0.8569 for first-order, 0.9137 for the Higuchi model, 0.8699 for the Hixson–Crowell model, and 0.2568 for Korsmeyer–Peppas model. The drug release mechanism of formulation F3 was found to follow the zero-order kinetics and Higuchi model. Additionally, the value of n obtained from the Korsmeyer–Peppas model was less than 0.45, indicative that the release pattern following the Fickian diffusion, signifying that the predominant drug release mechanism is by diffusion.

### 3.9. Antimicrobial Test

**Figure 4:**
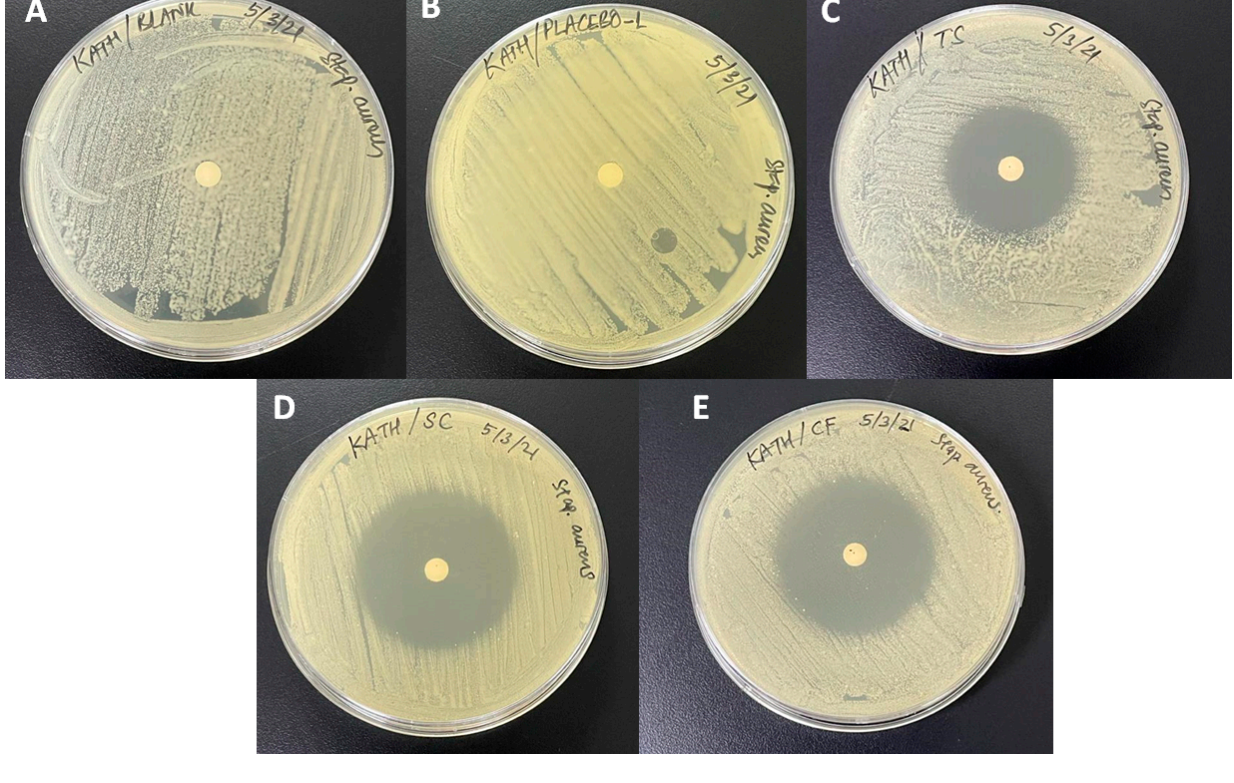
Antimicrobial test results by disc diffusion method ((**A**) controlled; (**B**) optimized formulation F3 without drug; (**C**) marketed clindamycin topical solution (1% *w*/*v*); (**D**) standard clindamycin solution (1% *w*/*v*); (**E**) formulation F3 with clindamycin (1% *w*/*v*)).

The therapeutic effectiveness of formation was determined by an antimicrobial test. Five samples including blank and placebo were prepared and tested with the disc diffusion method, using three plates for each sample. The measurement of the zone of inhibition was taken in triplicate for each plate to obtain the average value. The optimized formulation (F3) showed an average zone of inhibition at 43.44 ± 1.34 mm, (Figure 4E) while the average zone of inhibition for a marketed 1% clindamycin topical solution tested was at 33.11 ± 1.17 mm (Figure 4C). A 1% standard clindamycin solution was also tested as a reference; the average zone of inhibition obtained was at 43.78 ± 1.67 mm (Figure 4D). Antimicrobial data demonstrated the significant potential of in situ gel formulation of clindamycin over the marketed formulations available for treatment.

### 3.10. Cytotoxicity Test

**Figure 5:**
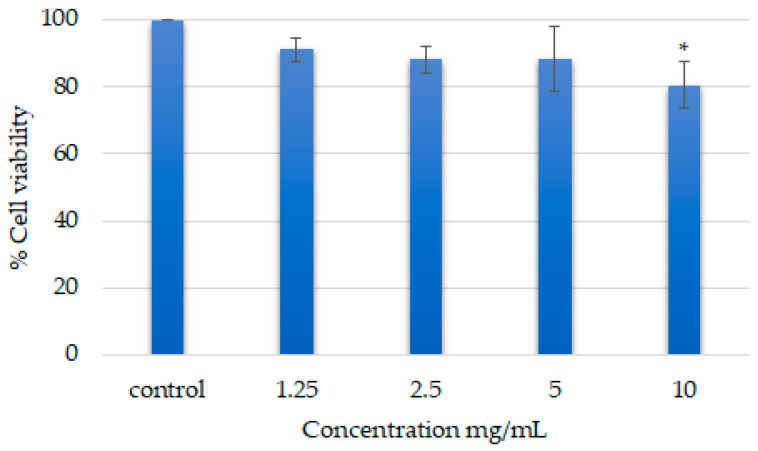
Concentration-dependent cytotoxicity test results for the optimized formulation (F3). Data presented as mean ± SD; *n* = 4; * indicates significant change in viability of the cells when compared to the control group (*p* < 0.05).

Safety of the formulation is the primary concerm, so in this regard, the cytotoxicity study of the optimized formulations (F3) was conducted on the HaCaT cell line. Different concentrations (1.25 mg/mL, 2.5 mg/mL, 5 mg/mL, and 10 mg/mL) of the optimized formulation on HaCaT cells were tested to observe the % cell viability. The results revealed that the viability of the 1.25 mg/mL, 2.5 mg/mL and, 5 mg/mL was 91.1 ± 3.6%, 88.2 ± 4.0%, and 88.1 ± 9.7%, respectively, considering 100% viability of the control cells. Finally, the highest concentration of the optimized formulation (10 mg/mL) showed a viability of 80.6 ± 6.9% (Figure 5). However, no significant concentration-dependant cytotoxic effects were observed.

### 3.11. Cell Morphology

**Figure 6:**
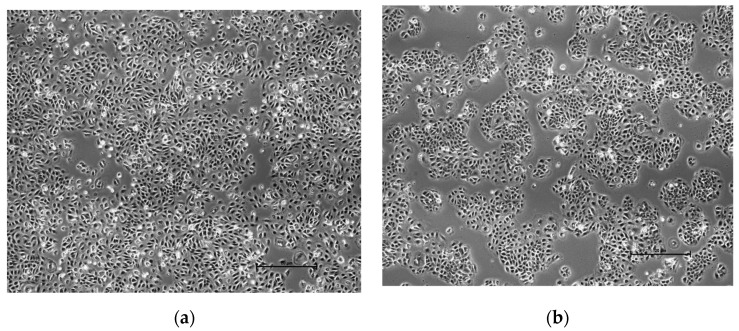
Morphology of human keratinocyte (HaCaT) cell line: (**a**) control cells, and (**b**) cells treated with gel (10 mg/mL) (F3) under microscope at 4×.

The cell’s size and shape were observed visually under the microscope (Nikon Eclipse Ti-U Inverted Microscope) at 4×. The results were focused on comparing the cells treated with the highest concentration (10 mg/mL) samples of the optimized formulation with the control. From the visual observation, there were no differences or changes found in the shape and size between the cells (Figure 6), which indicates the formulation is safe to use for clinical purpose.

## 4. Discussion

From the literature reviewed, there have been limited studies regarding topical formulations that could be effective in the treatment of HS. Clindamycin has been proven to be the only antibiotic effective in the treatment of HS via randomized controlled clinical trials [21,22]. The marketed topical products containing clindamycin are in solution and gel form, but they are meant to focus more on treating acne than the long-term inflammation associated with HS on the skin of patients. Moreover, there are some limitations observed on the conventional dosage forms, for instance, the necessity of frequent administration, especially for the drug with short half-life, fluctuations of the drug levels [23], as well as the higher chance of cross-contamination on the applicator. The approach for this study is to consider a formulation that could possess bioadhesion and a sustained-release effect to enhance the residence time for the drug at the site of skin, as the inflammation associated with HS stage I and mild stage II is mainly present at the epidermis and upper dermis layer [8]. There has also been an effective treatment shown for the oral clindamycin in combination with rifampicin, but topical medications could avoid the possible irritation of the gastrointestinal tract (GI); moreover, this could prevent the complex metabolism of the drug happening within the liver, which would then lead to an increment of bioavailability of the drug. HS is categorized as an autoinflammatory keratinization disease, in which topical drug delivery could initiate the therapeutic effect directly as soon as it acts upon the site of inflammation [24].

In the past, in situ gelling systems have been of great interest as the mucoadhesive drug delivery system where these delivery systems had shown to have the suitability of sustained drug release [25]. These days, in situ gelling systems have also been proven to ease the convenience of drug delivery for local applications as they can be easily administered onto the site of affected area [26]. Furthermore, the use of stimuli of the site of application has been explored with the possibilities of fabricating in situ formulations by the use of various polymers [27]. As our formulation is intended to be used topically, the most important stimuli to pay attention to in in situ gel formation is the temperature of the skin. It is expected to obtain an in situ spray that could exert an immediate sol–gel transition effect when the droplet dispensed come into contact with the human skin. A combination of both natural and synthetic polymers can be used in the formulation to achieve the ideal condition. Some of the main advantages of in situ gel formulations include a sustained-release drug delivery system, ease of administration, reduction in the frequency of application, improvement in steady levels [23], and moreover, the prevention in cross-contamination as the formulation would be administered in spray form, meaning no contact was necessary on the applicator.

Pluronic F-127, as categorized in a family containing more than 30 ABA-type triblock copolymers [28,29] with the components of 70% polyoxyethylene units and 30% polyoxypropylene blocks, is found to be the most commonly used polymer in pharmaceuticals due to its varied nature, one of the most important characteristics being the ability to form a thermoreversible gel at the temperature close to body temperature [29,30,31]. It has been suggested that the effect of thermogelation is due to the dehydration at the critical micelle temperature of polyoxypropylene (PPO) blocks. The increase in temperature would give a more significant result until the association of micelles occurs with each other at a specific temperature to form a gel. Moreover, another hypothesis has also been made suggesting that as the temperature increases, the modification of hydration spheres around the hydrophobic units could also induce the interactions along with the intramolecular hydrogen bonding effect [29,30,31]. From the results obtained, the average gelation temperature and gelation time decrease as the concentration of Pluronic F-127 increases, which leads to higher concentration of PPO responsible for the gelation effect. Based on the literation, lower Pluronic F-127 concentration can form a gel at a longer time; however, a concentration above 22% of Pluronic F-127 becomes gel at room temperature that is not considerable for spray formulation [32,33]. The requirement for the gelation temperature of the base formulation was set between 29 °C and 35 °C, and the gelation time was set at less than 2.30 min. The results have been tested by the ANOVA post-hoc Tukey test and the concentration of Pluronic F-127 at 18% and 19% showed no significant difference, hence 18% Pluronic F-127 was taken for further consideration.

Pluronic F-127 has the ability to assemble itself into polymeric micelles as well as various structures depending on the quality of the solvent, the critical micelle concentration, and the critical micelle temperature as it exists in various compositions. The hydrophilic shell supports its stability in an aqueous state, and under certain conditions, the hydrophobic core of Pluronic F-127 could take over for a temporal period to form a gel as a consequence of disorder-order transition that occurs in micelle packing [34,35]. Pluronic F-127 is considered a potential polymer because of its biocompatibility, which has been one of the most studied polymers in recent formulation development [34]. Although Pluronic F-127 could be used to prolong the residence time of the drug formulation [29], the maximum contact time, for instance in the human eye, was observed to last for just 1 h; in addition, one of the main drawbacks for Pluronic F-127 was its poor mechanical strength [30]. Therefore, to improve mechanical strength and adhesion time a combination of bioadhesive polymer with pluronic were used. As shown in the results, this addition resulted in the reduction of gelation temperature and gelation time. This phenomenon could be attributed to the gelation effect of Pluronic F-127, which occurs through H-bonding between the oxygen atoms of the ether group with water. Thus, an addition of compounds containing hydroxyl groups that enhance H-bonding could result in increased viscosity as well as gel strength [30]. From the studies and the results analyzed, a few bioadhesive polymers were then selected to formulate along with Pluronic F-127. The intention was to achieve an optimized formulation with an optimum gel strength and bioadhesion, to improve the contact time between the formulation and skin, as well as to possess a good sustained-release effect for the drug-loaded condition later on.

The concentrations of polymers are crucial on the effects of residence time, viscosity, as well as the release of the drug within the gelling system [9]. HPMC and SA have been decided as the bioadhesive polymers to be used in the development of the formulation. Due to its good biocompatibility and biodegradability along with wide availability and ease of use [36], HPMC has been a popular ingredient as a drug delivery matrix in the form of a film or gel for controlling the release of both hydrophilic and hydrophobic drugs [37]. A study had also been concluded that Pluronics are compatible to develop a safe and stable temperature-based in situ gelling system with bioadhesive properties when combined with HPMC [35].

On the other hand, as an anionic copolymer composed of 1,4-linked β-D-mannuronic acid (M-blocks) and α-L-guluronic acid (G-blocks) [38], SA is categorized as one of the natural polymers often used in drug delivery systems in various pharmaceutical formulations, which also include the targeted or localized drug-delivery systems [39]. SA was found to have potential in the extension of drug release [30] as it is bioadhesive, biocompatible, and biodegradable [40]. It is widely used as a coating element of drugs to facilitate the controlled release of active substances [41]. Another interesting point of SA includes the ability to resolve inflammation characteristics in the healing process [41]. However, there have always been studies suggesting combining the alginate with other biopolymers [41,42] or synthetic polymers to improve limitations, such as low stability.

Suitable analysis had been performed for the desired concentration of Pluronic F-127, ranging from 17% to 22% with HPMC and SA separately, where each of the bioadhesive polymers was kept constant at 0.2%. The results showed a clear inverse correlation relationship for both the polymers formulated with an increase in concentrations of Pluronic F-127. As the concentrations of Pluronic F-127 increased, the gelation temperature as well as gelation time for the formulations with both the polymers tested decreased. The decrease in gelation temperature was due to the interaction between the hydrophobic portions of the polymer molecule; this could have caused the disruption of micelle structure, thus increasing the entanglement of the micelle [11]. The main principle is the change in temperature responsible for the formation of gel, due to the presence of Pluronic F-127. The polymer chains form hydrogen bonds with the water molecules as they remain water soluble below the lower critical solution temperature. As the temperature increased, hydrogen bonds break and the domination of hydrophobic associations occurred, hence resulting in the gel formation [11]. A sustained drug delivery system can mainly depend upon the use of these types of polymers with the ability to undergo sol-gel transition at physiological conditions, or simply known as the human body temperature [43].

A variation of concentrations of HPMC and SA were then used to formulate with 18% Pluronic F-127 to test for the gelation temperature and gelation time. The concentrations used were 0.1%, 0.2%, and 0.4%. By visual inspection, it was found that the total concentration of HPMC and SA used in the formulation of the in situ spray should not exceed 0.4%, otherwise the formulation may be too viscous for the spraying. The results have shown that 0.2% SA has a great influence on reducing the gelation temperature as well as gelation time when added to the formulation consisting of 18% Pluronic F-127 with HPMC, at both the 0.1% and 0.2% concentrations. This indicates that the addition of SA results in aiding the formulations to reach their gel state faster. A study had also been conducted and proved that as the concentration of bioadhesive polymer increased, the gelation temperature decreased; hence, the time taken for gel formation was observed to be decreased. A similar pattern was reported by Ur-Rehman et al. and Chaudhari et al. in their research [44,45].

The optimum pH value of the optimized formulation was set in the range of pH 5.5–7.0 for the skin-friendly characteristic. The pH of the optimized formulation was recorded at pH 5.8, suitable for the skin. Compared to the base formulation of 18% Pluronic F-127 solution, the viscosity increased with the presence of bioadhesive polymer; and the highest viscosity was observed for the formulation with a combination of both the bioadhesive polymers (0.2% HPMC and 0.2% SA). The results showed about 43 times more viscosity as the formulation at body temperature converts to the gel when compared to the corresponding formulation at 25 °C. The formulation was in its solution form at 25 °C; when the surrounding temperature of the formulation reached 37 °C, transition to gel was formed and hence high viscosity was increased.

Bioadhesion is the adherence of two components with each other for a longer period by the influence of the interfacial force of attraction [46]. Bioadhesive strength was measured by the tensile-strength measurement [40], where tensile strength is the force required to detach the adhesive bond between the formulation and skin. A standard condition has been applied for the adhesion to occur using 0.5 N force for 20 s. Hydrophilic polymers possess excellent bioadhesive properties as they contain carboxylic groups. Both HPMC and SA have been classified as having an excellent bioadhesive property [40]. The results also showed that the bioadhesive polymers used, HPMC and SA, had a direct correlation with the tensile strength, and hence supported better bioadhesion property as compared to the base formulation containing no additional polymers other than Pluronic F-127. The results have shown the tensile strength of 18% Pluronic F-127 alone in formulation F, with 0.2% HPMC in formulation F1, with 0.2% SA in formulation F2, the combination of 0.2% HPMC and 0.2% SA in F3, and the combination of 0.1% HPMC and 0.2% SA in F4.

A comparison has been made between the formulations F3 and F4 with tensile strengths of 0.80 ± 0.041 N and 0.59 ± 0.020 N, respectively, to know if there is any significant difference, as the gelation temperature and gelation time, 29.0 ± 0.50 °C at 1.35 ± 0.40 min for F3, and 29.3 ± 0.29 °C at 1.48 ± 0.20 min for F4, showed no significant difference. As analyzed by the ANOVA post-hoc Tukey test, there is a significant difference shown between the formulation F3 and F4 for the bioadhesion test. It was found that HPMC held an important role, being one of the bioadhesive polymers in the formulation to help enhance bioadhesion. One of the most important factors that determined the increase in duration of action was the suitable ratio of polymers [46]. A study was also found to prove the point that, as the concentration of HPMC increased, the bioadhesive strength also increased [35].

The retention test, observed by spraying the formulations onto the fresh pigskin kept constant at 37.0 ± 0.2 °C, mainly focused to mimic the axilla, showing a direct correlation with the bioadhesion strength. The greater the tensile strength recorded, the faster the gel formed on the skin, and the longer it stayed intact. The gel was not affected by the room temperature at all, even after 2 h. Three of the formulations, F1, F2, and F3, were tested where the results showed that F1 and F2 took approximately the same period to set as gel, while F3 was noticed to have slightly lesser time taken for the gelation to occur. This had further proved the strength of the combination of bioadhesive polymers compared to the non-combination formulations. One of the most important phenomena for the sustained-release dosage form includes the dissolution and release of drugs. Bioadhesive polymers play major roles in sustaining the drug release [35]. SA can provide a sustained release for highly water-soluble drugs [47]. A formulation containing a combination of alginate and HPMC was observed to retain the drug better than the alginate or HPMC solutions alone, with over 10 h of in vitro-sustained drug release effect [30]. The combination of polymers has been suggested for developing in situ drug delivery systems as it could reduce the requirement on the amount of polymers for gelation and achieve better gels with improved gelling properties [30]. The results have shown a good, sustained drug release effect for the optimized formulation (F3) with 65.05% cumulative release in 8 h, whereas compared to the marketed topical solution, it showed a burst release of 84.30% within 1 h, and the marketed topical gel completed its drug release of 82.58% within 3 h.

The drug release of thermoreversible gels is influenced by various important factors; this includes the solubility of the drug within polymer and water, water diffusion rate into the polymer gel, the drug diffusion rate from the polymer gel, and the dissolution of the polymer within the experimental conditions [17]. Additionally, the hydrophobicity attributed to polymer synthesis and processing could be one important influencing factor of drug release from the thermosensitive in situ gelling system [48]

The relationship between drug release and the models is based on a few main factors such as the particle size distribution, the physical state of the drug within the formulation, the dissolution and diffusion properties of the loaded drug, and the viscoelastic properties of the polymer-penetrant system. The mathematical modelings play a significant role in facilitating the development of the formulations by helping to understand the drug release behavior in various dosage forms [49]. The drug release kinetics of the optimized formulation (F3) was found to fit best to the Higuchi model with R2 equal to 0.9137, and secondly to the zero-order kinetics at 0.8939. As Higuchi is known as a diffusion-based model [50], the result has suggested that the drug release of the optimized formulation (F3) is mainly by diffusion, further supported by the Fickian diffusion determined from the value of n in Korsmeyer–Peppas’ model as well. The n value based on the Korsmeyer–Peppas model obtained for the optimized formulation (F3) was at 0.2568.

From the results obtained, the zone of inhibition of *Staphylococcus aureus* was significantly greater for the optimized formulation (F3) as compared to the marketed topical solution with an equal amount of 1% clindamycin exposed. There is a significant difference shown between the marketed product (C) and the optimized formulation (E) as analyzed using an ANOVA post-hoc Tukey test, with the *p*-value obtained at 0.0001.

From the cytotoxicity test conducted and evaluated by MTT assay for cell viability of the optimized formulation (F3) without clindamycin loaded, with the concentration as high as 10.0 mg/mL of the formulation used, the percentage cell viability 72 h post-treatment was shown to maintain a good condition for the viable cells at the percentage of more than 80%, which is higher than the threshold value of 50%. There were no significant differences between the treated cells at different concentrations, indicative as no direct cytotoxic effects were observed for almost all the concentrations (1.25 mg/mL, 2.5 mg/mL, 5 mg/mL). The treated cells were observed under the microscope and the results showed no changes in shape and size despite the concentration at 10.0 mg/mL; this indicated that the optimized formulation (F3) is compatible with skin and it is safe to use [20,51,52]. The reason why the cytotoxicity test was not carried out along with the addition of clindamycin into the formulation is because clindamycin has already been known as a safe drug to use with no side effects towards human skin with the concentration of less than or equal to 2%; hence, the main focus of this was to test if the optimized formulation (F3) was compatible for the skin by assessing the direct cytotoxic effects. HaCaT cells were used as the representation of human skin in this study. The conversion of MTT into purple-colored formazan by viable cells would be the indicator, with the percentage cell viability measured with microplate reader at 570 nm and 630 nm. The lighter the purple color in the results after adding the MTT reagent following incubation for 4 h, the more cells were alive after the treatment [53].

## 5. Conclusions and Future Recommendation

Following several evaluations on the formulations with different bioadhesive polymers, an optimized in situ spray for local delivery of antibacterial drug intended for use on early stage HS condition was developed using Pluronic F-127 as the thermo-reversible gelling polymer, with bioadhesive polymers HPMC and SA. The formulation with this combination of polymers performed superior results to achieve the optimum bioadhesion and sustained-release property. The findings were better than the formulations with individual polymers. The viscosity showed that the gel of the optimized formulation (F3) showed approximately 43 times more viscosity at 37 ± 0.2 °C than the solution formed within the constant room temperature. This was further indicated that the formulation is suitable for use as in situ gelling system. Moreover, the initial pH value of the formulation was found to be acceptable with skin compatibility range and even closer to the average skin’s natural pH of 5.5. The in situ spray was found to convert the formulation to gel immediately once sprayed onto the ex vivo fresh pigskin. The optimized in situ spray was found to offer sustained release of the drug with its in situ gel property when adhered on the topical site. This could enhance patients’ compliance. The usage of bioadhesive polymers in the formulation ensures the improvement in residence time of the formulation along with the gel formed. This prolongs the treatment time and reduces the frequency of reapplication. Furthermore, the optimized formulation showed no cytotoxic effects, which demonstrates that there will be no risk of skin irritation even with the long contact time of up to 10 h. The availability of an effective topical formulation specially intended for the treatment of HS would provide a superior tool to prevent the possible suffering of HS patients in the future. There are, however, certain limitations in our study. As the methodologies were performed in vitro and ex vivo, further in vivo studies are required to confirm the effectiveness of the optimized formulation.

## Figures and Tables

**Table 1:** Presentation of gelation temperature and gelation time of different Pluronic F-127 aqueous dispersions.

Concentration of Pluronic F-127 (%*w*/*v*)	Gelation Temperature (°C) (Mean ± SD)	Gelation Time (min) (Mean ± SD)	pH
14	No gelation		6.634
16			6.758
17	34.0 ± 1.08	3.21 ± 0.01	6.774
18	31.2 ± 0.58	2.22 ± 0.02	6.832
19	29.0 ± 1.00	1.43 ± 0.02	6.851
20	28.0 ± 0.50	1.25 ± 0.05	6.908
22	26.7 ± 0.76	1.12 ± 0.10	7.043

(Data presented as mean ± SD; *n* = 3).

**Table 2:** Presentation of gelation temperature and gelation time of different combinations of bioadhesive polymers and Pluronic F-127.

Concentration of Pluronic F-127 (%*w*/*v*)	Conc. of HPMC (%*w*/*v*)	Conc. of SA (%*w*/*v*)	Gelation Temperature (°C) (mean ± SD)	Gelation Time (min) (mean ± SD)	pH
17	0.2	-	32.3 ± 0.30	2.35 ± 0.30	6.218
18	0.2	-	29.2 ± 0.29	1.55 ± 0.70	6.322
19	0.2	-	28.0 ± 0.76	1.33 ± 0.10	6.439
20	0.2	-	27.5 ± 0.50	1.12 ± 0.30	6.508
22	0.2	-	25.7 ± 0.28	0.52 ± 0.40	6.628
17	-	0.2	No gelation		6.224
18	-	0.2	28.8 ± 0.79	2.37 ± 0.50	6.346
19	-	0.2	27.2 ± 0.29	2.14 ± 0.60	6.445
20	-	0.2	26.0 ± 0.50	1.28 ± 0.20	6.513
22	-	0.2	24.5 ± 0.50	0.55 ± 0.70	6.631

(Data presented as mean ± SD; *n* = 3).

**Table 3:** Optimization of combinations of bioadhesive polymers and Pluronic F-127.

Concentration of Pluronic F-127 (%*w*/*v*)	Conc. of HPMC (%*w*/*v*)	Conc. of SA (%*w*/*v*)	Gelation Temperature (°C) (Mean ± SD)	Gelation Time (min) (Mean ± SD)	pH
18	0.1	-	29.8 ± 0.58	2.13 ± 0.20	6.328
18	0.2	-	29.2 ± 0.29	1.55 ± 0.70	6.322
18	0.4	-	26.7 ± 0.28	1.23 ± 0.40	6.314
18	-	0.1	29.0 ± 0.50	2.44 ± 0.20	6.339
18	-	0.2	28.8 ± 0.79	2.37 ± 0.50	6.346
18	-	0.4	25.5 ± 0.87	2.25 ± 0.50	6.358
18	0.1	0.1	29.7 ± 0.29	2.23 ± 0.30	6.336
18	0.1	0.2	29.3 ± 0.29	1.48 ± 0.20	6.341
18	0.2	0.1	29.2 ± 0.28	2.11 ± 0.60	6.332
18	0.2	0.2	29.0 ± 0.50	1.35 ± 0.40	6.343

(Data presented as mean ± SD; *n* = 3).

**Table 4:** Presentation of calculated ex vivo bioadhesion results of different formulations.

Formulation Name	Concentration of Pluronic F-127 (%*w*/*v*)	Concentration of HPMC (%*w*/*v*)	Concentration of Sodium Alginate (%*w*/*v*)	Bioadhesion (Newton) (Mean ± SD)
F	18	-	-	0.42 ± 0.012
F1	18	0.2	-	0.53 ± 0.031
F2	18	-	0.2	0.57 ± 0.019
F3	18	0.2	0.2	0.80 ± 0.041
F4	18	0.1	0.2	0.59 ± 0.020

(Data presented as mean ± SD; *n* = 3).

## Data Availability

Data are freely available.

## References

[1] Kassab Y., Muhamad S., Aldahoul H., Mohammed I., Paneerselvam G., Ayad M. (2019). The impact of skin disorders on patients’ quality of life in Malaysia. J. Clin. Intensive Care Med..

[2] Szepietowski J.C., Matusiak Ł. (2020). Hidradenitis Suppurativa: The Disease Which Stimulates Researchers and Clinicians. Dermatology.

[3] Ring H.C., Riis Mikkelsen P., Miller I.M., Jenssen H., Fuursted K., Saunte D.M., Jemec G.B. (2015). The bacteriology of hidradenitis suppurativa: A systematic review. Exp. Dermatol..

[4] Kokolakis G., Wolk K., Schneider-Burrus S., Kalus S., Barbus S., Gomis-Kleindienst S., Sabat R. (2020). Delayed Diagnosis of Hidradenitis Suppurativa and Its Effect on Patients and Healthcare System. Dermatology.

[5] Ferris A., Harding K. (2019). Hidradenitis suppurativa: A clinical summary. Wounds UK.

[6] Scuderi N., Monfrecola A., Dessy L.A., Fabbrocini G., Megna M., Monfrecola G. (2017). Medical and Surgical Treatment of Hidradenitis Suppurativa: A Review. Ski. Appendage Disord..

[7] Napolitano M., Megna M., Timoshchuk E.A., Patruno C., Balato N., Fabbrocini G., Monfrecola G. (2017). Hidradenitis suppurativa: From pathogenesis to diagnosis and treatment. Clin. Cosmet. Investig. Dermatol..

[8] Nomura T. (2020). Hidradenitis Suppurativa as a Potential Subtype of Autoinflammatory Keratinization Disease. Front. Immunol..

[9] Dasankoppa F.S., Solankiy P., Sholapur H.N., Jamakandi V.G., Sajjanar V.M., Walveka P.M. (2017). Design, formulation, and evaluation of in situ gelling ophthalmic drug delivery system comprising anionic and nonionic polymers. Indian J. Heal. Sci. Biomed. Res..

[10] Kurniawansyah I.S., Sopyan I., Aditya W.A., Nuraini H., Dwi Alminda F., Nurlatifah A. (2018). Preformed gel vs in situ gel: A review. Int. Res. J. Pharm..

[11] Lakshmi P., Harini K. (2019). Design and Optimization of Thermo-reversible Nasal in situ Gel of Atomoxetine Hydrochloride Using Taguchi Orthogonal Array Design. Dhaka Univ. J. Pharm. Sci..

[12] Hurler J., Škalko-Basnet N. (2012). Potentials of Chitosan-Based Delivery Systems in Wound Therapy: Bioadhesion Study. J. Funct. Biomater..

[13] Aslani A., Ghannadi A., Najafi H. (2013). Design, formulation and evaluation of a mucoadhesive gel from *Quercus brantii* L. and coriandrum sativum L. as periodontal drug delivery. Adv. Biomed. Res..

[14] Chin L.Y., Tan J.Y.P., Choudhury H., Pandey M., Sisinthy S.P., Gorain B. (2021). Development and optimization of chitosan coated nanoemulgel of telmisartan for intranasal delivery: A comparative study. J. Drug Deliv. Sci. Technol..

[15] Gupta A., Garg S., Khar R. (1993). Measurement of bioadhesive strength of mucoadhesive buccal tablets: Design of an in-vitro assembly. Indian Drugs.

[16] Asmani F., Abdullah I., Khan J., Budiasih S. (2015). Determination of Permeation Pathways of Clindamycin Phosphate into the Skin. Am. J. Pharm. Tech. Res..

[17] Fazli N., Khan A., Khan A., Nasir F., Iqbal Z., Khan I., Khan J.A., Khuda F., Zakir S., Yousaf N. (2013). Development and evaluation of pluronic-and methylcellulose-based thermoreversible drug delivery system for insulin OrBito IMI Project View project Taste masking View project Development and evaluation of pluronic-and methylcellulose-based thermoreversible. Drug Dev. Ind. Pharm..

[18] Purushothama P.S., Nayak M., Bhargav H.S., Shastri S.D., Nayak M.M. Measurement of the Zone of Inhibition of an Antibiotic. Proceedings of the 2016 IEEE 6th International Conference on Advanced Computing.

[19] Petushkova N.A., Rusanov A.L., Pyatnitskiy M.A., Larina O.V., Zgoda V.G., Lisitsa A.V., Luzgina N.G. (2020). Proteomic characterization of HaCaT keratinocytes provides new insights into changes associated with SDS exposure. Biomed. Dermatol..

[20] Riss T.L., Moravec R.A., Niles A.L., Duellman S., Benink H.A., Worzella T.J., Minor L. (2016). Cell Viability Assays.

[21] Hilton A., Armstrong R. (2006). Stat note 6: Post hoc anova tests. Microbiologist.

[22] Frew J.W., Hawkes J.E., Krueger J.G. (2019). Topical, systemic and biologic therapies in hidradenitis suppurativa: Pathogenic insights by examining therapeutic mechanisms. Ther. Adv. Chronic Dis..

[23] Mandhar P., Joshi G. (2015). Development of Sustained Release Drug Delivery System: A Review. Asian Pac. J. Health Sci..

[24] Rupal J., Mallikarjuna Setty C., Patel D., Kaushal J., Mallikarjuna S.C., Dipti P. (2010). Preparation and Evaluation of Topical Gel of Valdecoxib. Artic. Int. J. Pharm. Sci. Drug Res..

[25] Sanjana A., Ahmed M.G., BH J.G. (2021). Preparation and evaluation of in-situ gels containing hydrocortisone for the treatment of aphthous ulcer. J. Oral Biol. Craniofacial Res..

[26] Patel P., Patel P. (2015). Formulation and evaluation of clindamycin HCL in situ gel for vaginal application. Int. J. Pharm. Investig..

[27] Chatterjee S., Hui P.C. (2019). Review of stimuli-responsive polymers in drug delivery and textile application. Molecules.

[28] Bodratti A.M., Alexandridis P. (2018). Formulation of Poloxamers for Drug Delivery. J. Funct. Biomater..

[29] Nagai N., Isaka T., Deguchi S., Minami M., Yamaguchi M., Otake H., Okamoto N., Nakazawa Y. (2020). In Situ Gelling Systems Using Pluronic F127 Enhance Corneal Permeability of Indomethacin Nanocrystals. Int. J. Mol. Sci..

[30] Jain D. (2016). Newer Trends in In Situ Gelling Systems for Controlled Ocular Drug Delivery. J. Anal. Pharm. Res..

[31] Yu J., Qiu H., Yin S., Wang H., Li Y. (2021). Polymeric Drug Delivery System Based on Pluronics for Cancer Treatment. Molecules.

[32] Cunha-Filho M.S.S., Alvarez-Lorenzo C., Martínez-Pacheco R., Landin M. (2012). Temperature-sensitive gels for intratumoral delivery of β-lapachone: Effect of cyclodextrins and ethanol. Sci. World J..

[33] Geng H., Song H., Qi J., Cui D. (2011). Sustained release of VEGF from PLGA nanoparticles embedded thermo-sensitive hydrogel in full-thickness porcine bladder acellular matrix. Nanoscale Res. Lett..

[34] Liu L., Yong K.-T., Roy I., Law W.-C., Ye L., Liu J., Liu J., Kumar R., Zhang X., Prasad P.N. (2012). Bioconjugated Pluronic Triblock-Copolymer Micelle-Encapsulated Quantum Dots for Targeted Imaging of Cancer: In Vitro and In Vivo Studies. Theranostics.

[35] Swamy G.N., Abbas Z. (2012). Mucoadhesive in situ gels as nasal drug delivery systems: An overview. Artic. Asian J. Pharm. Sci..

[36] da Silva Júnior W.F., de Oliveira Pinheiro J.G., Moreira C.D.L.F.A., de Souza F.J.J., de Lima Á.A.N. (2017). Alternative Technologies to Improve Solubility and Stability of Poorly Water-Soluble Drugs. Multifunctional Systems for Combined Delivery, Biosensing and Diagnostics.

[37] Kaur G., Grewal J., Jyoti K., Jain U.K., Chandra R., Madan J. (2018). Oral controlled and sustained drug delivery systems: Concepts, advances, preclinical, and clinical status. Drug Targeting and Stimuli Sensitive Drug Delivery Systems.

[38] Shivakumara L.R., Demappa T. (2019). Synthesis and Swelling Behavior of Sodium Alginate/Poly(vinyl alcohol) Hydrogels. Turkish J. Pharm. Sci..

[39] Zhang H., Cheng J., Ao Q. (2021). Preparation of Alginate-Based Biomaterials and Their Applications in Biomedicine. Mar. Drugs.

[40] Kumar K., Dhawan N., Sharma H., Vaidya S., Vaidya B. (2014). Bioadhesive polymers: Novel tool for drug delivery. Artif. Cells Nanomed. Biotechnol..

[41] Puscaselu R.G., Lobiuc A., Dimian M., Covasa M. (2020). Alginate: From Food Industry to Biomedical Applications and Management of Metabolic Disorders. Polymers.

[42] Hariyadi D.M., Islam N. (2020). Current status of alginate in drug delivery. Adv. Pharmacol. Pharm. Sci..

[43] Nguyen D.D., Lai J.-Y. (2020). Advancing the stimuli response of polymer-based drug delivery systems for ocular disease treatment. Polym. Chem..

[44] Chaudhari S.D., Misra M., Kumar Mandal U. (2010). Formulation and evaluation of thermoreversible mucoadhesive microemulsion based in-situ gel (TMMIG) of an anti-osteoporotic agent. Artic. J. Glob. Pharma Technol..

[45] Ur-Rehman T., Tavelin S., Gröbner G. (2011). Chitosan in situ gelation for improved drug loading and retention in poloxamer 407 gels. Int. J. Pharm..

[46] Dasankoppa F.S., Kujur S., Sholapur H.P.N.A., Jamakandi V.G. (2016). Design, formulation and evaluation of carboxy methyl tamarind based in situ gelling ophthalmic drug delivery system of dorzolamide hydrochloride. Indian J. Heal. Sci. Biomed. Res..

[47] Thulluru A., Mohan Varma M., Setty C.M., Chintamaneni P.K., Samayamanthula S. (2015). Effect of Sodium alginate in Combination With HPMC K 100 M in Extending the Release of Metoprolol Succinate from its Gastro-Retentive Floating Tablets. Indian J. Pharm. Educ. Res..

[48] Zahir-Jouzdani F., Wolf J.D., Atyabi F., Bernkop-Schnürch A. (2018). In situ gelling and mucoadhesive polymers: Why do they need each other?. Expert Opin. Drug Deliv..

[49] Padmaa Paarakh M., Ani Jose P., Setty C.M., Christoper G.V.P. (2018). Release kinetics—Concepts and applications. Int. J. Pharm. Res. Technol..

[50] Guo P., Buttaro B.A., Xue H.Y., Tran N.T., Wong H.L. (2020). Lipid-polymer hybrid nanoparticles carrying linezolid improve treatment of methicillin-resistant *Staphylococcus aureus* (MRSA) harbored inside bone cells and biofilms. Eur. J. Pharm. Biopharm..

[51] Boateng J., Okeke O. (2019). Evaluation of Clay-Functionalized Wafers and Films for Nicotine Replacement Therapy via Buccal Mucosa. Pharmaceutics.

[52] Patel N., Lalwani D., Gollmer S., Injeti E., Sari Y., Nesamony J. (2016). Development and evaluation of a calcium alginate based oral ceftriaxone sodium formulation. Prog. Biomater..

[53] Mohamed M.A., Nasr M., Elkhatib W.F., Eltayeb W.N. (2018). In Vitro Evaluation of Antimicrobial Activity and Cytotoxicity of Different Nanobiotics Targeting Multidrug Resistant and Biofilm Forming Staphylococci. Biomed Res. Int..

